# On the Reliability of Switching Costs Across Time and Domains

**DOI:** 10.3389/fpsyg.2018.01032

**Published:** 2018-06-22

**Authors:** Kalinka Timmer, Marco Calabria, Francesca M. Branzi, Cristina Baus, Albert Costa

**Affiliations:** ^1^Center for Brain and Cognition, Pompeu Fabra University, Barcelona, Spain; ^2^Neuroscience and Aphasia Research Unit, School of Biological Sciences, University of Manchester, Manchester, United Kingdom; ^3^Institució Catalana de Recerca i Estudis Avançats, Barcelona, Spain

**Keywords:** bilingual language control, executive control, test–retest reliability, cross-talk, switching costs

## Abstract

Bilingual speakers are suggested to use control processes to avoid linguistic interference from the unintended language. It is debated whether these bilingual language control (BLC) processes are an instantiation of the more domain-general executive control (EC) processes. Previous studies inconsistently report correlations between measures of linguistic and non-linguistic control in bilinguals. In the present study, we investigate the extent to which there is cross-talk between these two domains of control for two switch costs, namely the *n-1 shift cost* and *the n-2 repetition cost*. Also, we address an important problem, namely the reliability of the measures used to investigate cross-talk. If the reliability of a measure is low, then these measures are ill-suited to test cross-talk between domains through correlations. We asked participants to perform both a linguistic- and non-linguistic switching task at two sessions about a week apart. The results show a dissociation between the two types of switch costs. Regarding test–retest reliability, we found a stronger reliability for the *n-1 shift cost* compared to the *n-2 repetition cost* within both domains as measured by correlations across sessions. This suggests the *n-1 shift cost* is more suitable to explore cross-talk of BLC and EC. Next, we do find cross-talk for the *n-1 shift cost* as demonstrated by a significant cross-domain correlation. This suggests that there are at least some shared processes in the linguistic and non-linguistic task.

## Introduction

How do bilingual speakers control their two languages to avoid linguistic confusion? Researchers argue that this is achieved by a set of control processes labeled bilingual language control (BLC) (Green, 1998). But, what is the nature of these control processes? It is debated whether or not BLC is an instantiation of the more domain-general executive control (EC) processes (Green, 1998; Dijkstra and van Heuven, 2002; Abutalebi and Green, 2007). One of the most frequently used tasks to measure BLC and EC abilities is the switching paradigm and its switch cost measures (*n-1 shift cost* and *n-2 repetition cost*). The extent to which this cross-talk between domains is present for switch costs is inconsistent and controversial (Garbin et al., 2010; Branzi et al., 2016a; Timmer et al., 2017). Here, we report a study that explores: (a) the cross-talk between the two systems by looking at switch costs in the two domains, and (b) the reliability of two switching measures often used to explore the cross-talk of control mechanisms between BLC and EC. We argue that the reliability of these measures varies considerably and that when they are reliable, cross-talk between domains is present.

The current evidence regarding cross-talk between BLC and EC comes from four sources. First, and especially relevant for our purposes, are studies that compared performance of bilinguals in linguistic and non-linguistic switching tasks. Most studies did not reveal a correlation of switch costs across domains (Calabria et al., 2011; Prior and Gollan, 2013; Cattaneo et al., 2015; Branzi et al., 2016a; Declerck et al., 2017; but see Declerck et al., 2015). Second, studies that compared brain activity for the switch cost in the two domains for the same bilinguals showed there is some degree of overlapping activation but also a contribution of different regions (De Baene et al., 2015; Weissberger et al., 2015; Branzi et al., 2016b; Timmer et al., 2017). Third, somewhat indirect evidence comes from studies comparing monolinguals and bilinguals. The hypothesis is that bilinguals have long-term practice in BLC which can affect the performance of EC. The results are mixed, while some studies show smaller behavioral switch costs on a non-linguistic task for bilinguals than monolinguals (Prior and Macwhinney, 2010; Prior and Gollan, 2011; Houtzager et al., 2017), many other studies did not (Hernández et al., 2013; Paap and Greenberg, 2013; Paap et al., 2017; Timmer et al., 2017; Branzi et al., 2018). Four, studies that show a relation between how much people switch between their languages on a daily basis and the switch cost in a non-linguistic task (Hartanto and Yang, 2016). Thus, the results regarding cross-talk for the switch cost is inconsistent and controversial.

However, one important problem when considering the above studies is the reliability of the measures used to investigate cross-talk. If reliability of a measure is low, then the absence of cross-talk between domains is uninformative. For example, if no correlation is observed for switch costs across domains, one might be tempted to conclude that there is no cross-talk for switching abilities in the two domains. However, before drawing such a conclusion we need to know whether the switch cost measures are reliable themselves within each domain. If there is poor test–retest reliability the measures do not consistently distinguish the performance of individuals within a population. This inability to distinguish between individuals makes these measures ill-suited to detect relationship with other constructs in cross-domain correlational studies (Hedge et al., 2017). Thus, with poor test–retest reliability the result is silent about the cross-talk between domains. In this study, we test the reliability of switch costs, one of the most frequently used measures regarding cross-talk of control processing, in a linguistic and non-linguistic switching task at two points in time with about a week in between.

This strategy has already been used in the context of EC measures (Miyake et al., 2000) where some measures of EC reached acceptable level of reliability whereas others did not (Williams et al., 2005; Beck et al., 2011; Willoughby and Blair, 2011; Paap and Sawi, 2016; Soveri et al., 2016; Donath et al., 2017; Fernández-Marcos et al., 2017). Of interest to the present study is that the switch cost measure (i.e., *n-1 shift cost*) during a non-linguistic task, as an index of EC efficiency, showed a reliable effect over time (*r* = 0.62) (Paap and Sawi, 2016). In addition, another type of switch cost, the *n-2 repetition cost* that reflects a differential process of inhibitory control (described underneath) did not show high reliability in non-linguistic switching tasks (between *r* = 0.23 and 0.44) (Pettigrew and Martin, 2016; Kowalczyk and Grange, 2017; Rey-Mermet et al., 2017). To the best of our knowledge, reliability for the language switch costs has not been tested. The present study extends the test of reliability to the language domain.

### Description of the Tasks and Measures

We asked participants to perform both a linguistic and non-linguistic switching task. In the linguistic switching task, participants named pictures in Catalan, Spanish, or English depending on the flag presented around the picture. In the non-linguistic switching task, participants made a decision about the color, size, or type (i.e., number/letter) of a visual stimulus depending on a visual cue presented around the stimulus.

These tasks were designed such that two types of switch costs could be measured: the *n-1 shift cost* and the *n-2 repetition cost*. The *n-1 shift cost* refers to the cost of switching between languages/tasks (switch trial; BA) compared to repeating the same language/task (repeat trial; AA). This cost is considered to measure people’s efficiency in applying transient control (Meiran, 2010; Bobb and Wodniecka, 2013). More specifically, it reflects cue encoding, the activation of a new set of S-R rules in working memory, and the inhibition of the previous task set (Periáñez and Barceló, 2009; Timmer et al., 2017, 2018). This measure has played an important role to inform theories of language control (Green, 1998; Roelofs, 1998; Costa et al., 1999; La Heij, 2005; Finkbeiner et al., 2006) and domain-general EC (Jurado and Rosselli, 2007; Munakata et al., 2011; Diamond, 2013).

The *n-2 repetition cost* refers to the cost of switching into a recently performed task (in an *n-2* trial) as compared to switching into a not-recently performed task. Consider a language switching task with three languages (Catalan, Spanish, and English) and the following language sequences (CBA and ABA). When participants name pictures in three different languages (CBA) each trial corresponds to a different language and hence no language repetition is present. However, in the sequence ABA the last instance (A) corresponds to the same language used two trials before. Comparing the performance in these two sequences is how the *n-2 repetition cost* is calculated, and this cost is often interpreted as cognitive processes that solve proactive interference (Mayr and Keele, 2000; Philipp et al., 2007; Branzi et al., 2016a). Thus, previous inhibition needs to be overcome to perform the current task suggesting this is a pure measure of inhibitory control (Mayr and Keele, 2000). While some suggest the *n-2 repetition cost* is a pure measure of inhibitory control (Philipp and Koch, 2009), others suggest that the *n-2* also measures other factors than only inhibition, for example episodic retrieval, and is therefore not a pure measure of one process (Grange et al., 2017; Kowalczyk and Grange, 2017).

Previous evidence regarding the cross-talk for these two costs are somewhat inconsistent. For example, correlations across domains did often not reveal cross-talk for either the *n-1 shift cost* (Calabria et al., 2011; Cattaneo et al., 2015; Branzi et al., 2016a; but see Declerck et al., 2015, 2017) or the *n-2 repetition cost* (Branzi et al., 2016a). But neural evidence suggests that there are some overlapping processes and regions underlying the switch costs (De Baene et al., 2015; Weissberger et al., 2015; Branzi et al., 2016b; Timmer et al., 2017). We investigate whether the absence of cross-domain correlations is caused by the lack of test–retest reliability of the switching measures in both the linguistic- and non-linguistic task. We expect to replicate the reliability of the *n-1 shift cost* during non-linguistic task switching (Paap and Sawi, 2016), while we expect to find lower reliability for the *n-2 repetition cost* (Pettigrew and Martin, 2016; Rey-Mermet et al., 2017). We extend these findings to the linguistic domain expecting to find similar results in the linguistic as in the non-linguistic domain.

## Materials and Methods

### Participants

Fifty-eight Catalan-Spanish-English trilingual from Universitat Pompeu Fabra were paid for their participation (35 females; average age: 23.3 years; *SD* = 4.12). They all had normal or corrected-to-normal vision, and no history of neurological impairments or language disorders. Four participants were excluded due to low accuracy on the linguistic switching task, for these participants less than 65% of trials were left due to high error rate and many voice-key errors. One participant was excluded due to technical failure of the voice key. The final sample consisted of 53 participants (32 females; average age: 23.5 years; *SD* = 4.22).

All participants completed a self-rating proficiency and social economic background questionnaire to assess their language proficiency and social-economic background. The language proficiency is reported in **Table 1**. The mothers’ [4.1 (*SE* = 1.08)] and fathers’ [4.0 (*SE* = 1.41)] education level were measured on a 6-point scale (1) primary school, (2) middle school, (3) high-school diploma, (4) professional training, (5) Bachelor University, and (6) Master or Ph.D.). They also performed the Superior Scale I of the Ravens Advanced Progressive Matrices to measure non-verbal intelligence. Participants had to indicate which of eight possible pieces was missing from a picture. Twelve picture items were tested (Raven et al., 1998). On average participants had a score of 9.5 (*SD* = 1.54) out of a maximum of 12.

**Table 1:** Mean answers (and standard deviations) to self-rating proficiency and social economic background questionnaire.

	Catalan	Spanish	English
Speaking^a^	6.8 (0.51)	6.5 (0.66)	5.0 (1.07)
Understanding^a^	7.0 (0.19)	6.9 (0.32)	5.7 (0.94)
Reading^a^	6.9 (0.26)	6.9 (0.29)	5.7 (0.89)
Writing^a^	6.7 (0.61)	6.6 (0.71)	5.0 (0.98)

*^a^A 7-point scale, with 1 point being the lowest proficiency and 7 being the highest self-rated proficiency*.

### Materials and Procedure

The experiment consisted of two sessions (test and retest) with approximately a week (5–9 days) between sessions. At both days the participants performed a linguistic- and non-linguistic switching task. The order in which the tasks were performed was counterbalanced across participants and kept in the same order over sessions for each participant. Each session took approximately 1.5 h during which they were seated individually in a quiet room with dimmed lights and seated approximately 1 m from the computer screen. At the first session, before starting the switching tasks, participants signed an informed consent form, filled out the language proficiency questionnaire, and completed the Raven’s non-verbal intelligence test before the experimental tasks (**Table 1**). Instructions for the switching tasks were given in oral and written format. They were instructed to make responses as fast and accurate as possible. They received practice trials before each task.

#### Linguistic Switching Task

In the linguistic switching task, participants named eight black-and-white drawings representing nouns with non-cognate names between Catalan (L1), Spanish (L2), and English (L3) (see Branzi et al., 2016a for the stimuli). The pictures were presented one at a time. Each picture was surrounded by four cue-signs (flag in the corners of the picture) indicating the language in which each picture was to be named (four Catalan, Spanish, or English flags). All target stimuli were centered and presented in black on a white background. The speech onset latencies were measured with a voice-key. Before the experiment, participants were familiarized with the pictures and their corresponding names in the three languages to make sure they produced the correct names for the pictures.

In total participants named 648 randomized pictures divided over six blocks. After each block participants could take a short break and start the next block when ready. Each trial started with the presentation of the cue-signs together with a tone. After 100 ms (CSI), the picture was presented in the middle of the screen while the cues also remained on the screen. Picture and cue-signs remained on the screen until a response was given. After each response a blank screen appeared before the next cue was presented for the following trial.

#### Non-linguistic Switching Task

In the non-linguistic switching task, participants made three perceptual classifications about visual stimuli. The three classifications were ‘color’ (red vs. blue), ‘size’ (small vs. big), and ‘type’ (letter vs. number) as used in previous studies (Philipp and Koch, 2006; Branzi et al., 2016a). Just as the flags during the linguistic task, cues were presented around the target stimulus to notify the classification to be made. For the ‘color’ decision the cue was a yellow square, for the ‘size’ decision the cue was an arrow pointing up and down, and for the ‘type’ decision the cue was a paragraph sign. Responses were given manually with key presses to three response keys for each hand. Note also that responses were labeled on the keyboard.

The procedure of the non-linguistic task was identical to that of the linguistic one. The only difference was that for the non-linguistic task participants received feedback on their performance, accuracy in % was shown at the end of each block, while no feedback was given on the linguistic task.

### Data Analysis

The experimental design was the same as previous studies investigating the *n-1 shift cost* and *n-2 repetition cost* (Philipp and Koch, 2006; Branzi et al., 2016a, 2018). Depending on the two preceding trials the current *n* trial was allocated to one of three conditions (CAA, CBA, and ABA). Each language (Catalan, Spanish, and English) or task (colors, size, and type) was assigned a letter (A, B, or C). Within each of the three conditions the latter letter refers to the current *n* trial. For example, for the CAA condition the *n* trial is A and preceded at the *n-1* trial by another A. Given that the preceding trial is identical to the current this is an *n-1 repetition* condition. For the CBA condition, the *n* trial A is preceded at the *n-1* trial by a B and at the *n-2* trial by a C. Both preceding trials are different from A and therefore considered an *n-2* switch condition. For the ABA condition, the *n* trial A is preceded at *n-1* by B, a different trial, but at *n-2* by A, a repetition of trial n. Here, at *n-2* there is a repetition of the *n* trial and therefore the condition is called *n-2* repetition.

The two effects are calculated by comparing two conditions. The *n-1 shift cost* is the difference between the RTs from CAA (*n-1* repetition) and CBA (*n-2* switch) conditions. The *n-2 repetition cost* is the difference between the RTs of the CBA (*n-2* switch) and the ABA (*n-2* repetition) conditions.

Importantly, it has been suggested that the *n-2 repetition cost* can be eliminated when the number of trials in all three conditions is equal. However, when the number of *n-1* repetition trials is greatly reduced, in comparison to the other two conditions, the *n-2 repetition cost* is present (see Philipp and Koch, 2006). As we are interested in both costs we have reduced the number of *n-1* repetition trials. Both the *n-2* repetition trials (ABA) and the n-switch trials (CBA) occurred approximately on 39% of the trials, while the *n-1* repetition trials (CAA) occurred on approximately 11% of the trials. The sum does not add up to 100% because the first two trials of each block were removed as well as the trial after a repetition trial (CAA). See Branzi et al. (2016a, 2018) for the same procedure and further details.

Non-linguistic and linguistic switching data were analyzed separately with a repeated measure ANOVA that included the within-subject factors Session (test vs. retest) and Trial type (CAA vs. CBA vs. ABA). This was followed by correlational analyses to examine, among others, the test–retest reliability between sessions. Bonferroni corrections for multiple comparisons are applied when necessary.

## Results

**FIGURE 1:**
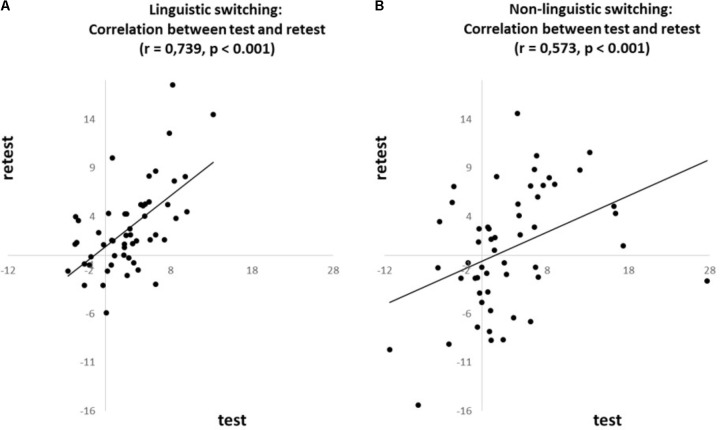
Correlations for the ***n-1 shift cost***, based on the proportional costs, between test and retest for **(A)** linguistic and **(B)** non-linguistic switching task.

**FIGURE 2:**
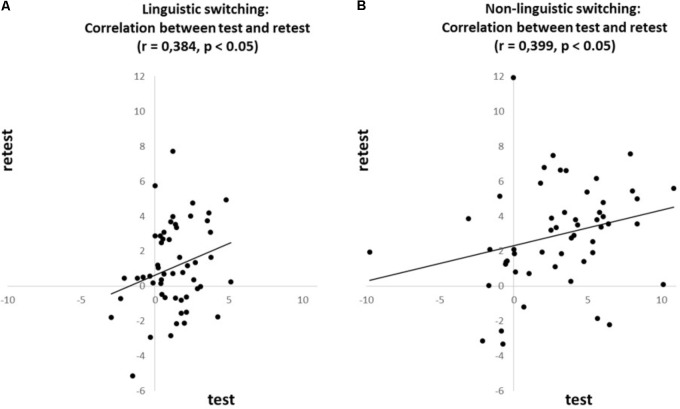
Correlations for the ***n-2 repetition cost***, based on the proportional costs, between test and retest for **(A)** linguistic and **(B)** non-linguistic switching task.

**FIGURE 3:**
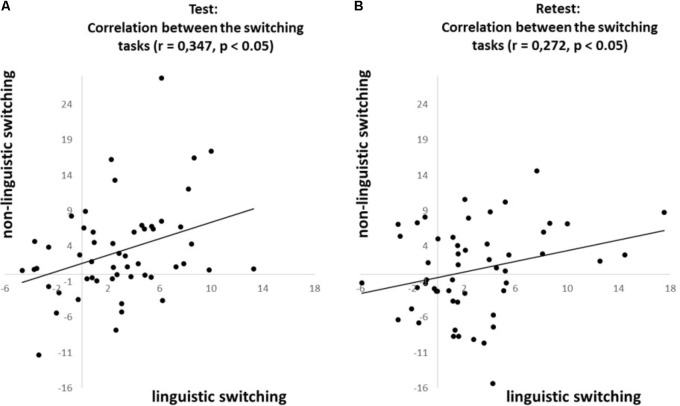
Correlations for the ***n-1 shift cost***, based on the proportional costs, between the linguistic and non-linguistic switching tasks at **(A)** test and **(B)** retest.

**FIGURE 4:**
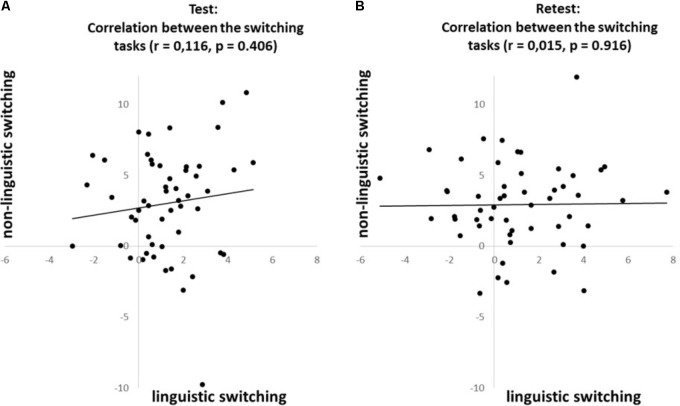
Correlations for the ***n-2 repetition cost***, based on the proportional costs, between the linguistic and non-linguistic switching tasks at **(A)** test and **(B)** retest.

We first present the response latency analyses separate for the two tasks (**Table 2**) and second the correlations within and between tasks (**Figures 1–4**).

**Table 2:** Mean response latencies in ms (and standard error) for the linguistic and non-linguistic switching tasks for each Trial type by Session, as well as the magnitude of the *n-1* *shift cost* and the *n-2* *repetition cost* in ms.

	Test			Retest			*n-1 shift cost*		*n-2 repetition cost*	
	CAA	CBA	ABA	CAA	CBA	ABA	Test	Retest	Test	Retest
Linguistic switching task	1097 (21.1)	1147(26.2)	1170 (27.2)	1039 (23.6)	1087 (29.5)	1106 (30.0)	51	48	23	18
Non-linguistic switching task	1072 (33.9)	1126 (37.4)	1177 (38.0)	846 (25.0)	859 (29.6)	897 (31.7)	54	13	50	38

### Linguistic Switching Task

Outliers were discarded from the analysis [naming latencies longer than 5,000 ms (0.2% of the data) and latencies that deviated 2.5 SD from the average per participant per condition (4.1% of the data)]. In addition, voice-key errors (1.5% of the data) and incorrect responses were also discarded (4.0% of the data). The first two trials after an error were also removed (7.2% of the data) as the Type of trial (CAA, CBA, or ABA) could not be determined until 2 n after an error. In the analysis a total of 83.5% of the trials was included at test and 82.7% at retest. No differences in accuracy were observed between Sessions or Trial types.

Naming latencies at retest were 61 ms faster than at test [*F*(1,52) = 15.75, *MSe* = 18666.82, *p* < 0.001, $\eta_{p}^{2}$ = 0.233]. The main effect of Trial type was also significant [*F*(1,104) = 37.24, *MSe* = 5949.21, *p* < 0.001, $\eta_{p}^{2}$ = 0.417], revealing the presence of both and *n-1 shift cost* and *n-2 repetition cost*. The *n-1 shift cost* was reflected by faster latencies for CAA than CBA trials [respectively, 1,068 ms and 1,117 ms; *F*(1,52) = 27.61, *MSe* = 9371.87, *p* < 0.001, $\eta_{p}^{2}$ = 0.347]. The *n-2 repetition cost* was reflected by faster latencies for CBA than ABA trials [respectively, 1,117 ms and 1,138 ms; *F*(1,52) = 26.91, *MSe* = 1669.11, *p* < 0.001, $\eta_{p}^{2}$ = 0.341]. There was no interaction between Session and Trial type (*F* < 1).

### Non-linguistic Switching Task

The same criteria to remove outliers and errors was used as in the linguistic task (latencies longer than 5,000: 0.5% of the data; 2.5 SD outliers: 3.6%; errors: 2.6%; 2 trials after error: 4.6%). In the analysis a total of 88% of the trials was included at test and 89.4% at retest. No differences in accuracy were observed between Sessions or Trial types.

Response latencies at retest were 257 ms faster than at test [*F*(1,52) = 182.49, *MSe* = 28886.72, *p* < 0.001, $\eta_{p}^{2}$ = 0.778]. The main effect of Trial type was also significant [*F*(1,104) = 33.45, *MSe* = 6387.06, *p* < 0.001, $\eta_{p}^{2}$ = 0.391], revealing the presence of both and *n-1 shift cost* and *n-2 repetition cost*. The *n-1 shift cost* was reflected by faster latencies for CAA than CBA trials [respectively, 959 ms and 993 ms; *F*(1,52) = 8.74, *MSe* = 13517.09, *p* < 0.005, $\eta_{p}^{2}$ = 0.144]. The *n-2 repetition cost* was reflected by faster latencies for CBA than ABA trials [respectively, 993 ms and 1037 ms; *F*(1,52) = 48.69, *MSe* = 4230.65, *p* < 0.001, $\eta_{p}^{2}$ = 0.484]. However Session and Trial type interacted [*F*(1,104) = 8.59, *MSe* = 2926.78, *p* < 0.001, $\eta_{p}^{2}$ = 0.142]. This showed that the size of the *n-1 shift cost* decreased significantly from test to retest [respectively, 54 ms and 13 ms; *t*(52) = 2.87, *SE* = 14.29, *p* < 0.01]. In contrast, the *n-2 repetition cost* did not decrease significantly over testing sessions [50 ms and 38 ms; *t*(52) = 1.24, *SE* = 9.96, *ns*].

### Correlations

For the correlations we calculated a proportional cost for each of the switch costs to avoid problems of differences between tasks in speed of responding. The switch cost was divided by the average RT of the involved trials and multiplied by a hundred.^1^

To investigate whether the *n-1 shift and n-2 repetition costs* are consistent over time we correlated [Intra Class Correlation (ICC); also named Cronbach’s alpha] each of these costs between test and retest. The *n-1 shift cost* revealed a positive correlation between test and retest for both the linguistic (*r* = 0.739, *p* < 0.001; see **Figure 1A**) and non-linguistic switching tasks (*r* = 0.573, *p* < 0.001; see **Figure 1B**). The *n-2 repetition cost* also revealed a test–retest correlation for both the linguistic (*r* = 0.384, *p* < 0.05; see **Figure 2A**) and non-linguistic switching tasks (*r* = 0.399, *p* < 0.05; see **Figure 2B**).

To investigate whether the *n-1 shift and n-2 repetition costs* are consistent across domains we correlated (Pearson’s coefficient) each of these costs between the linguistic- and non-linguistic switching tasks. The *n-1 shift cost* revealed a positive correlation across domains at both test (*r* = 0.347, *p* < 0.05; see **Figure 3A**) and retest (*r* = 0.272, *p* < 0.05; see **Figure 3B**). In contrast, the *n-2 repetition cost* does not reveal correlations across domains at neither test (*r* = 0.116, *ns*; see **Figure 4A**) nor retest (*r* = 0.015, *ns*; see **Figure 4B**).

## Discussion

We explored the test–retest reliability of linguistic and non-linguistic switch costs (*n-1 shift and the n-2 repetition cost*), as well as the presence/absence of cross-talk between the two cognitive control domains for both switch costs. Participants performed switching tasks in both domains at two sessions, approximately a week apart. The current study revealed a dissociation between the two types of switch costs (*n-1 shift cost* and *n-2 repetition cost*) regarding their test–retest reliability and the cross-talk between domains. The test–retest reliability for the *n-1 shift cost* was quite high as the correlation between sessions indicates, both in the linguistic and non-linguistic tasks. However, this reliability was much lower for the *n-2 repetition cost*. This pattern indicates that *the n-1 shift cost* is more stable across time than the *n-2 repetition cost*, and consequently the former is more suitable to explore whether there are correlations across domains that would suggest shared constructs. Cross-talk between the two domains was present for the *n-1 shift cost* as demonstrated by a cross-domain correlation. This suggests that there are at least some shared processes in the linguistic and non-linguistic task.

We looked at the correlations of the proportional switch costs instead of the mean RTs as the latter will often show high reliability due to overlapping processes (e.g., perceiving the visual stimulus and the motor processing of pressing a button or making a vocal response) in the RTs of switch and repeat trials (Declerck et al., 2015). In addition, the difference scores reflect a specific process within switching paradigms (Miller and Ulrich, 2013). The present study revealed weaker test–retest correlation for the *n-2 repetition cost* than the *n-1 shift cost*. Thus, in the present study the *n-2 repetition cost* does not rank individuals consistently, either due to high error variance or due to low between-subjects variance. The inability to distinguish between individuals makes this measure ill-suited to investigate shared constructs across domains (Hedge et al., 2017). To conclude, when investigating questions of cross-talk with correlational paradigms it is advisable to use the *n-1 shift cost* and be careful with the use of the *n-2 repetition cost*.

Next to increased error variance and low between-subjects variance, it is to be noted that practice effects can also diminish the test–retest reliability of a measure (Paap and Sawi, 2016). Performance on a simple choice RT task improves over time in speed and accuracy. Some part of this practice effect is removed by a short practice at the beginning of the experiment, however, there is still a practice effect across testing days. While both switch costs were present at first testing in both domains, the *n-1 shift cost* decreased from test to retest for the non-linguistic task but not for the linguistic task. Thus, these differential practice effects depending on the domain in which the switching paradigm was conducted can diminish the reliability over time.

While the *n-1 shift cost* showed good test–retest reliability, the *n-2 repetition cost* only showed a weak reliability for both domains. This is in line with previous studies investigating the *n-2 repetition cost* in the non-linguistic domain (Pettigrew and Martin, 2016; Kowalczyk and Grange, 2017; Rey-Mermet et al., 2017). Therefore, no conclusion can be drawn about the convergent validity across domains for the *n-2 repetition cost*. In light of low reliability, we did not find a cross-domain correlation either for the *n-2 repetition cost*, in line with Branzi et al. (2016a). The absence of reliability could be due to the fact that some have suggested that the *n-2 repetition cost* is not a pure measure of one process, inhibition, but is also influenced by factors like episodic memory (Grange et al., 2017; Kowalczyk and Grange, 2017). If this cost arises due to a mixture of underlying measures it is not strange that the reliability is low. In addition, it has been suggested that this measure might have different underlying processes in each domain and that these processes do not vary in the same way across the two tasks. For example, the linguistic task showed variations in the mechanisms of the *n-2 repetition cost* depending on which of the three different languages was used (Babcock and Vallesi, 2015). This shows that this measure is more complex than assumed within the linguistic domain and does not have a direct relation to the non-linguistic domain. Thus, the present study shows that the reliability of the *n-2 repetition cost* is weak over time and therefore no conclusions can be drawn on whether there is cross-talk across domains for this measure.

For the *n-1 shift cost*, there was strong test–retest reliability and we also find a cross-domain correlation suggesting that the mechanisms underlying the *n-1 shift cost* share at least some processes in the linguistic and non-linguistic task. Note that a correlation of 0.6 is often considered to reflect a good reliability within the literature (Landis and Koch, 1977; Cicchetti and Sparrow, 1981), however, there are no definitive guidelines on how to interpret correlational values (Crocker and Algina, 1986). Not all studies showed a relation between the linguistic and non-linguistic task for this measure (Calabria et al., 2011; Prior and Gollan, 2013; Calabria et al., 2015; Cattaneo et al., 2015; Branzi et al., 2016a; Declerck et al., 2017). This could potentially be due to a couple of reasons. First, the test–retest reliability observed for this switch cost in each domain limits the correlation that can be observed between them. While we have strong reliability for the *n-1* shift-cost, there is always some measurement error and the cross-domain correlation can never be higher than the test-reliability of both measures. The magnitude of the cross-domain correlation is attenuated by measurement error of both measures. This can have impact on theoretical conclusions, where non-significant correlational results are interpreted as an absence of shared constructs across domains, though there might be shared constructs that are not picked up due to high measurement errors (Hedge et al., 2017).

Second, we investigate the switch cost together with the *n-2 repetition cost*. To show effects on the latter cost the number of repeat trials was greatly reduced compared to the other trial types (Philipp and Koch, 2006), while previous studies had an equal number of switch and repeat trials (but see Branzi et al., 2016a). This could have changed the mechanism measured in the *n-1 switch cost* as participants might use a different strategy within such a set-up of trials.

Third, other paradigms have suggested there is some but not full overlap across domains for the switch cost (Green, 1998; Dijkstra and van Heuven, 2002; Abutalebi and Green, 2007; Grainger et al., 2010; Declerck et al., 2015). For example, studies that compared brain activity in linguistic versus non-linguistic switching tasks directly found only some overlapping areas to be activated (De Baene et al., 2015; Weissberger et al., 2015; Branzi et al., 2016b) and electrophysiological comparisons only showed the P3 but not the N2 component to overlap for the switch cost (Timmer et al., 2017). This suggests that the switch cost reflects a multitude of underlying processes that may differ to a certain extent depending on the domain (Declerck et al., 2015). For example, the stimuli used in each task are often different (e.g., pictures versus alphabetic and numerical representations). Also, the modality of response is different (oral naming vs. categorization). The difference in the response-set is important on two points. First, manual responses are more diverse than speech responses. For speech production there is only one output through the vocal tracts, while manual responses involve completely different responses (e.g., left vs. right hand response). In the present study the oral response is in one of three languages, but the manual response is one of six buttons. Second, the underlying processes that accumulate to an oral or manual response develop differently over time. Competing representations and responses start diverging at a later point in time for speech production than for manual responses as has been observed by ERPs and impact the behavioral responses differentially (Tillman and Wiens, 2011; Acheson et al., 2012; Timmer and Chen, 2017). The final performance (size of the switch cost) of an individual is affected by all sub-processes: those shared between tasks and those that have differential contributions (Declerck et al., 2017). Therefore, it is possible that most studies do not reveal correlation due to the sub-processes that differ, making it difficult to detect the contribution of possible shared processes. But due to the common sub-process the correlation might sometimes present regardless of the variation in other sub-processes. However, a conclusion of some shared sub-processes needs to be taken with caution.

## Conclusion

To conclude, test–retest reliability for the *n-1 shift cost* is strong in both the non-linguistic and the linguistic domain, therefore, the *n-1 shift cost* is stable and can be used to test convergent validity across domains. In contrast, the reliability for the *n-2 repetition cost* that measures a different process was weaker, therefore the *n-2 repetition cost* should be used with caution when investigating correlations regarding cross-talk. While the *n-1 shift cost* seems to have at least some shared processes in the linguistic and non-linguistic domain, no conclusions can be drawn regarding the *n-2 repetition cost*.

## Ethics Statement

The study was approved by the ethical committee board at Universitat Pompeu Fabra. All participants were adults aged 18 or more. At the beginning of the experimental session participants signed an informed consent form that stated a description of the experiment and stressed that the participant is free to leave the experiment at any time without providing any explanation to the experimenter. If the participant wants to proceed, they sign the consent form and the experiment commences.

## Author Contributions

All authors substantially contributed to the conception or design of the manuscript, interpretation of the data for the manuscript, and revising the manuscript critically. KT and FB contributed to the data acquisition. KT contributed to the analysis of the data and critical drafting and revising of the manuscript. All authors are in agreement to be accountable for all aspects of the work in ensuring that questions related to the accuracy or integrity of any part of the work are appropriately investigated and resolved.

## Conflict of Interest Statement

The authors declare that the research was conducted in the absence of any commercial or financial relationships that could be construed as a potential conflict of interest.

## Footnotes

1 Note that the correlational analyses based on the original mixing and switch costs show a similar pattern of results as the analyses based on the proportional costs reported here, and the same conclusions would be drawn as described in the text.
